# Towards a more ambitious mental health agenda for Scotland

**DOI:** 10.1371/journal.pmen.0000366

**Published:** 2025-07-28

**Authors:** Martyn Pickersgill

**Affiliations:** Centre for Biomedicine, Self and Society, The Usher Institute, The University of Edinburgh, Usher Building, Edinburgh, United Kingdom; PLOS: Public Library of Science, UNITED KINGDOM OF GREAT BRITAIN AND NORTHERN IRELAND

On the 6 May 2025, the First Minister of Scotland, John Swinney, set out a new Programme for Government (PfG) [1]. The National Health Service (NHS) Scotland is front and centre, with the third paragraph of Swinney’s Foreword noting investments to enhance healthcare accessibility. Regarding mental health specifically, the PfG states: “Over the last year, we’ve seen […] faster support for children needing mental health support” (p30). Related commitments are to “Improving mental health support for young people – clearing Child and Adolescent Mental Health Services backlogs and meeting the 18-week standard nationally by December 2025 – backed by £123.5 million recurring funding for mental health” (p32).

Adult mental health will be served by, for instance, providing “access to digitally enabled psychological interventions and therapies for people who may benefit from early treatment”. Further, “the Communities Mental Health and Wellbeing Fund for Adults [CMHW Fund] will entail a further commitment of £30m funding until 2027” (p32). The PfG also states a focus on “Reducing mental health demands on police officers and protect[ing] people in crisis, including improving frontline training and the development of national guidance to improve multi-agency working when responding to a person experiencing a psychiatric [sic] emergency” (p32).

While welcome, the PfG mental health commitments lack ambition. The CMHW Fund, for example, aims to: “Support community based initiatives that promote and develop good mental health and wellbeing and/or mitigate and protect against the impact of distress and mental ill health within the adult population (aged 16 or over), with a particular focus on prevention and early intervention” (p3) [2]. Given £30m equates to less than £7 per individual over the age of 16 living in Scotland, it is unclear whether these aims can be met. This unfortunately reflects a tendency internationally to under-invest in public mental health beyond treatment per se (which is itself hardly well-funded) [3].

In terms of frontline services, it appears that much of the significant current need for clinical care will be tackled by “recurring” rather than additional funds. Some of the shortfall in service provision will, it seems, be addressed via digital interventions. In other words, it is challenging to locate any promises within the PfG about significantly more funding for mental health services and staffing. Again, this roll-out of digital mental health to increase access mirrors developments in a range of other countries [4]. Yet, concerns continue to circulate that the limits of digital interventions are insufficiently considered, nor are the efforts required to integrate them within healthcare systems [5].

There are, then, some issues with the 2025 PfG for Scotland in relation to mental health. These reflect and contribute to a larger concern about the finances that are available for healthcare, and the associated challenges of insufficient resourcing. Going forward, much more money is needed for such public mental health and wellbeing initiatives in Scotland (and, of course, beyond) [6]. It is important to reiterate this point in a context where money is regularly noted by politicians to be in ever shorter supply. A reluctance to assert the need for additional investment risks substantiating a discourse of inevitability, with the result that the under-funding of mental healthcare remains the status quo [7].

Money alone, though, is essential yet insufficient. To accompany the spending increases that are needed, a more ambitious overarching approach to mental health is also required. In terms of service-delivery specifically, Health Boards (i.e., the regional NHS Scotland bodies that deliver care) must be financially empowered to act in more devolved and imaginative ways with regards to how they provide care. In particular, Boards should be encouraged and facilitated to engage even more closely with communities.

Community collaboration is often regarded as paying dividends for healthcare, alongside addressing democratic goals [8]. To power collaboration, however, an increase in both direct resource and more symbolic recognition and valuation of this is required. Doing so is necessary for supporting NHS staff (including via protected time) to engage with communities to (re)design services that people want, and for which access is enhanced. In turn, the labour of individuals and communities to enhance the NHS must also be recognised.

Scotland’s geography is highly diverse, and its population increasingly so as well. Accordingly, local ingenuity and creativity must be more actively celebrated and enabled to be the norm. Different Boards have different challenges and opportunities, and would profit from even greater attention to the geographic, demographic, and socio-economic specificities of the localities within Board areas. Many NHS staff already undertake outreach and engagement with the communities they work with. Yet, such dialogical local adaptation can be driven by the kinds of compassion and civic mindedness that political pressures and financial constraints render evermore challenging.

To achieve these collaborative aims, and to better comprehend what collaboration can enable, Scottish policymakers and clinicians could afford benefit from additional learning from peers beyond those based in neighbouring countries like England. In India, for instance, the ways in which non-profit mental health organisations embed forms of collaborative governance could inform how community knowledge and leadership can be built into service design and delivery [9]. Research conducted in Chile, for example, illustrates how administrative and bureaucratic processes can shape yet also stabilise community collaboration [10]. Other work undertaken in South Africa has explored how context-specific interventions can draw on community insights to inform *where* interventions should be delivered [11].

These are but indicative examples; nonetheless, they underscore how mental healthcare innovation with the Global South provides a substantial evidence-base upon which Scotland should draw [12]. In turn, a more collaborative, devolved agenda for Scotland could be leveraged by other nations to drive forward their own resonant policy ambitions. Scotland is also an excellent ‘laboratory’ for more contextualised services: small enough to be nimble, rich enough to take risks, and with the policy, NHS, and material infrastructures to scaffold both pilot and more programmatic healthcare developments.

Within Scotland, an approach to service delivery that more purposefully builds in flexibility and collaboration is clearly required. Yet, such an ethos of reconfiguration, alongside an ethic of access, requires political ambition. It also demands a greater transfer of funds and power from politicians to Health Boards to clinicians and, indeed, to communities themselves.
